# Data from calorimetric decay heat measurements of five used PWR 17x17 nuclear fuel assemblies

**DOI:** 10.1016/j.dib.2019.104917

**Published:** 2019-12-02

**Authors:** Peter Jansson, Martin Bengtsson, Ulrika Bäckström, Kjell Svensson, Mattias Lycksell, Anders Sjöland

**Affiliations:** a Uppsala University, Sweden; b Swedish Nuclear Fuel and Waste Management Company, Sweden

**Keywords:** Spent nuclear fuel, Decay heat, Calorimetry, Calorimetric measurement

## Abstract

Raw data from calorimetric measurements of five nuclear fuel assemblies of the PWR 17 × 17 type are provided. Measurements of the temperature both inside a calorimeter, in which the fuel assembly was placed, as well as outside, were performed as a function of time while water circulating inside the calorimeter heats up from radiation emitted in the radioactive decay of material in the fuel assembly. The data contain also measurements of dose rate in the water outside the calorimeter. Data from 38 measurements using an electrically heated model of a fuel assembly are also provided to be used for, e.g., calibration.

The data can be used for validation of computer codes for modelling of nuclear systems, e.g. nuclear reactors, storage and transport of nuclear fuel or systems for geological disposal.

**Table:** Specifications Table

Subject	Nuclear Energy and Engineering
Specific subject area	High-level radioactive waste management
Type of data	Table
How data were acquired	Measurements of water temperature, both inside and outside of the calorimeter, were performed using PT-100 thermocouples 1/10 DIN 5929370-001 from Pentronic AB. Measurements of gamma dose rate outside the calorimeter was performed using EFD 999-600L devices from Scandiatronix Medical AB. Measurements of electric power consumption was performed using a 7600 ION device from Power Measurement LTD.
Data format	Raw
Parameters for data collection	Data were collected from the calorimeter at a pool temperature of about 19° Centigrade.
Description of data collection	After placing a used nuclear fuel assembly in the calorimeter, the circulating water was cooled to a few degrees below the surrounding pool temperature. After cooling, the decay heat from the fuel assembly generated by decay of unstable isotopes heats the water in the calorimeter. Measurements of the temperatures inside and outside of the calorimeter were performed together with measurements of the gamma dose rate outside the calorimeter. For the dose rate measurements, only sensor number three was calibrated. All measured data were logged as a function of time since start of the measurement. Measurements using an electrically heated model of a fuel assembly, where the power consumption was monitored, were performed in order to allow calibration between heat input rate and temperature increase rate.
Data source location	Institution: Central interim storage for spent nuclear fuel (Clab) City/Town/Region: Simpevarp/Oskarshamn Country: Sweden
Data accessibility	With the article

**Table:** 

**Value of the Data** • Calorimetric measurements of decay heat generated by nuclear fuel assemblies are very rare. They can be used, besides the explicit measurements of the heat source, to validate calculations of decay heat and provide feedback to needed adjustments of nuclear data (e.g. fission yields, Q values) used for calculations of decay heat and burnup. • High-level radioactive waste managing organisations, nuclear data evaluators, reactor physics calculators. • These data can be used as a basis for knowledge on how to design an experiment where decay heat of high-level radioactive waste, e.g. used nuclear fuel, is to be measured. Design of future experiments, including prioritizing which nuclear data to measure, can be based on results from validation of models against data provided here. • Public access to similar data is extremely rare. By providing this data, it can used to further enhance the capabilities of computer codes used for modelling nuclear systems, e.g. nuclear reactors, storage and transport systems or systems for geological disposal

## 1 Data

Data are stored as Excel spreadsheets with one measurement of one fuel assembly per file. The name of the file indicates which fuel assembly that was measured and on which date it was measured. The first rows of the spreadsheet file contain Meta information about the measurement. Measurement data are stored in columns, with data from each measurement sensor in each column, and with the time of the measurement increasing downwards in the spreadsheet.

Burnup, cooling time and initial enrichment of U-235 of the measured fuel assemblies are listed below.

**Table:** 

Fuel assembly	Burnup [MWd/kgU]	Cooling time at the time of the calorimetric measurement [y]	Initial U-235 enrichment [%]
BT01	52.7	4.5	3.95
BT02	55.1	8.6	3.95
BT03	50.3	9.8	3.95/2.80 (burnable absorber)
BT04	50.8	13.5	3.70
BT05	50.3	21.4	3.60

Data from the calibration measurements have the same structure as the measurements with nuclear fuel. The name of the file indicates that it is a calibration measurement if it begins with the word ‘EV’ and contains also the set electric power input as well as the measurement date.

Sensors named in the data, and what they are measuring, are listed as follows.

**Table:** 

Sensor name	Unit	Measurement
_251KA901_A_kW	Watt	Power input to the electrically heated model, if applicable.
_251KA509	Degree C	Temperature difference between inlet and outlet of the circulation flow through the calorimeter.
_251KA501_B1	Degree C	Temperature of the water in the inlet to the calorimeter.
_251KA501_B2	Degree C	Temperature of the water in the inlet to the calorimeter.
_251KA502_B1	Degree C	Temperature of the water in the outlet from the calorimeter.
_251KA502_B2	Degree C	Temperature of the water in the outlet from the calorimeter.
_251KA511_B1	Degree C	Temperature of the water inside the calorimeter.
_251KA511_B2	Degree C	Temperature of the water inside the calorimeter.
_251KA511_B3	Degree C	Temperature of the water inside the calorimeter.
_251KA511_B4	Degree C	Temperature of the water inside the calorimeter.
_251KA511_B5	Degree C	Temperature of the water inside the calorimeter.
_251KA511_B6	Degree C	Temperature of the water inside the calorimeter.
_251KA511_B7	Degree C	Temperature of the water inside the calorimeter.
_251KA511_B8	Degree C	Temperature of the water inside the calorimeter.
_251KA511	Degree C	Temperature of the water inside the calorimeter.
_251KA751_Vikt	Kilogram	The weight of an external tank with water, used to monitor that the fuel is constantly cooled.
_251KA101	Bar	The pressure in the inlet pipe to the calorimeter.
_251KA521_B1	Degree C	Temperature on the inside wall of the calorimeter.
_251KA522_B1	Degree C	Temperature on the outside wall of the calorimeter.
_251KA531_B1	Degree C	Temperature in the pool outside the calorimeter.
_251KA531_B2	Degree C	Temperature in the pool outside the calorimeter.
_251KA701	Gray/hour	Gamma dose rate outside the calorimeter.
_251KA702	Gray/hour	Gamma dose rate outside the calorimeter.
_251KA703	Gray/hour	Gamma dose rate outside the calorimeter.
_251KA704	Gray/hour	Gamma dose rate outside the calorimeter.
_251KA705	Gray/hour	Gamma dose rate outside the calorimeter.
_251EA1	Watt	Power input to the whole system.
EA1_Börvärde	Watt	The setpoint of power consumption of the electrically heated model, if applicable.
_251KA901_B_PA	Watt	Power input to the water circulation pump.

## 2 Experimental design, materials, and methods

Measurements where performed using the calorimeter described in more detail in Ref. [1]. Essentially, a closable stainless steel container (calorimeter) is used as a device to measure the increase of the temperature inside the container. The data provided can be evaluated by, e.g., the methods described in Refs. [2,3]. Each measurement was performed as follows:

1. Place a nuclear fuel assembly in the calorimeter and close it.
2. Cool the water in the calorimeter down to a few degrees below the temperature of the surrounding pool water.
3. Stop cooling and let the decay heat produced by the nuclear fuel heat up the calorimeter.
4. During the whole time, measure the temperatures both inside and outside of the calorimeter as well as dose rate in the water outside.
5. When the temperature in calorimeter has increased beyond the pool temperature by several degrees, stop the measurement and remove the fuel assembly.
